# Phylogenomic Resolution of the Cetacean Tree of Life Using Target Sequence Capture

**DOI:** 10.1093/sysbio/syz068

**Published:** 2019-10-21

**Authors:** Michael R McGowen, Georgia Tsagkogeorga, Sandra Álvarez-Carretero, Mario dos Reis, Monika Struebig, Robert Deaville, Paul D Jepson, Simon Jarman, Andrea Polanowski, Phillip A Morin, Stephen J Rossiter

**Affiliations:** 1 School of Biological and Chemical Sciences, Queen Mary University of London, Mile End Road, London E1 4NS, UK; 2 Department of Vertebrate Zoology, Smithsonian Museum of Natural History, 10th & Constitution Ave. NW, Washington DC 20560, USA; 3 Institute of Zoology, Zoological Society of London, Outer Circle, London NW1 4RY, UK; 4 School of Biological Sciences, University of Western Australia, 35 Stirling Highway, Perth WA 6009, Australia; 5 Australian Antarctic Division, 203 Channel Highway, Kingston TAS 7050, Australia; 6 Southwest Fisheries Science Center, National Marine Fisheries Service, NOAA, 8901 La Jolla Shores Dr., La Jolla CA 92037 USA

## Abstract

The evolution of cetaceans, from their early transition to an aquatic lifestyle to their subsequent diversification, has been the subject of numerous studies. However, although the higher-level relationships among cetacean families have been largely settled, several aspects of the systematics within these groups remain unresolved. Problematic clades include the oceanic dolphins (37 spp.), which have experienced a recent rapid radiation, and the beaked whales (22 spp.), which have not been investigated in detail using nuclear loci. The combined application of high-throughput sequencing with techniques that target specific genomic sequences provide a powerful means of rapidly generating large volumes of orthologous sequence data for use in phylogenomic studies. To elucidate the phylogenetic relationships within the Cetacea, we combined sequence capture with Illumina sequencing to generate data for $\sim $3200 protein-coding genes for 68 cetacean species and their close relatives including the pygmy hippopotamus. By combining data from $>$38,000 exons with existing sequences from 11 cetaceans and seven outgroup taxa, we produced the first comprehensive comparative genomic data set for cetaceans, spanning 6,527,596 aligned base pairs (bp) and 89 taxa. Phylogenetic trees reconstructed with maximum likelihood and Bayesian inference of concatenated loci, as well as with coalescence analyses of individual gene trees, produced mostly concordant and well-supported trees. Our results completely resolve the relationships among beaked whales as well as the contentious relationships among oceanic dolphins, especially the problematic subfamily Delphinidae. We carried out Bayesian estimation of species divergence times using MCMCTree and compared our complete data set to a subset of clocklike genes. Analyses using the complete data set consistently showed less variance in divergence times than the reduced data set. In addition, integration of new fossils (e.g., *Mystacodon selenensis*) indicates that the diversification of Crown Cetacea began before the Late Eocene and the divergence of Crown Delphinidae as early as the Middle Miocene. [Cetaceans; phylogenomics; Delphinidae; Ziphiidae; dolphins; whales.]

Cetaceans (whales, dolphins, and porpoises) have undergone the most dramatic morphological transformation of all mammals, having originated from a clade of terrestrial even-toed ungulates $>$50 Ma (Gatesy and O’Leary 2001). The origin and evolution of cetaceans have emerged as a textbook case for macroevolution and are arguably one of the best examples of morphological transition in the fossil record (Thewissen et al. 2009). Numerous remarkable fossils from the Eocene (56–34 Ma) have documented this seemingly insurmountable transition from land to sea, detailing such adaptations as the reduction of the hind limbs, reconfiguration of the spine, movement of the nostrils posteriorly, and development of underwater hearing (Berta et al. 2015; Marx et al. 2016).

After their transition to the sea, cetaceans further diversified into two groups with unique adaptations. Toothed whales (Odontoceti) acquired echolocation to hunt using ultrasonic pulses and a highly specialized inner ear, whereas baleen whales (Mysticeti) lost their teeth and evolved a novel keratinous material for filtering aggregate prey (Gatesy et al. 2013). Modern extant cetaceans number 89 recognized species, including 75 odontocetes and 14 mysticetes. These species have achieved a cosmopolitan distribution, living in tropical, temperate, and polar marine waters with some species exclusively inhabiting estuaries and river systems (Jefferson et al. 2015; Society for Marine Mammalogy Committee on Taxonomy 2017). Many cetaceans also possess other distinctive specializations, including reduced olfactory and gustatory capacity, the ability to see in dim light, large brains, enormous body size, extended longevity, complex social behavior, osmoregulatory innovations, and respiratory and circulatory systems for extended dives, all of which have made them supremely adapted to their aquatic environment (Gatesy et al. 2013; McGowen et al. 2014; Berta et al. 2015).

Although the evolution of cetaceans from an even-toed “ungulate” ancestor is well understood, there are aspects of their systematics that have proven more challenging. This is particularly the case for relationships within cetacean families, some of which remain problematic (Hamilton et al. 2001; McGowen et al. 2009; Steeman et al. 2009; Chen et al. 2011; Geisler et al. 2011; Zhou et al. 2011; Hassanin et al. 2012). For example, the most speciose cetacean family, Delphinidae (oceanic dolphins, $\sim $37 species), has been especially difficult to resolve despite recent attempts, likely due to this group’s recent rapid radiation (Leduc et al. 1999; Nishida et al. 2007; Caballero et al. 2008; McGowen et al. 2009; McGowen 2011; Amaral et al. 2012; Perrin et al. 2013). Particular confusion surrounds the phylogenetic relationships among $\sim $14 species of bottlenose-like dolphins (subfamily Delphininae); these radiated within $\sim $5 myr by some estimates (e.g. McGowen et al. 2009; Steeman et al. 2009; Slater et al. 2010) and little consensus exists among data sets, possibly due to incomplete lineage sorting, introgression, hybridization (either ancient or ongoing), and the slow mutation rate in cetaceans (Fig. 1; Kingston et al. 2009; McGowen et al. 2009; McGowen 2011; Amaral et al. 2012; 2014; Perrin et al. 2013).

**Figure 1.**
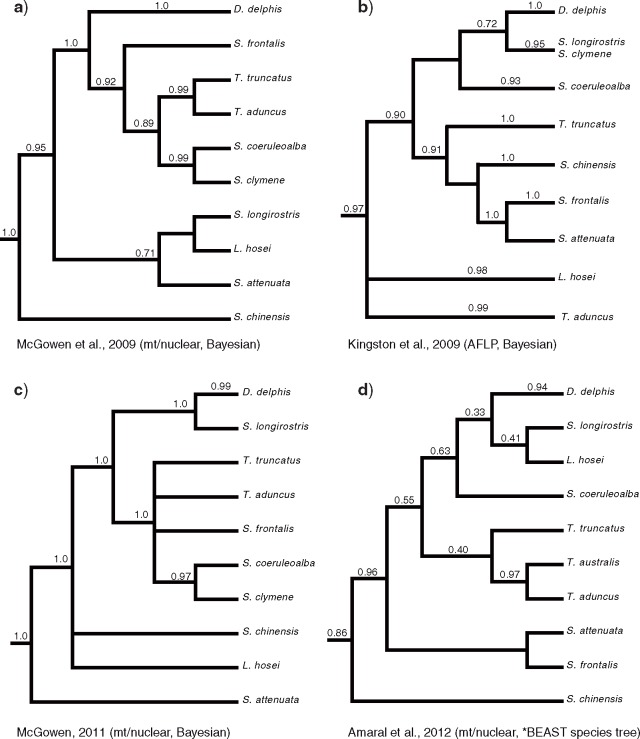
Four representative recent phylogenetic analyses of Delphininae (a–d) showing the disagreement in relationships between studies. All numbers above nodes represent Bayesian PP. Multiple individuals for species in Kingston et al. (2009) have been condensed into single OTUs, but support values have been retained.

Aside from oceanic dolphins, the relationships among taxa within two other speciose clades, the Balaenopteroidea (rorquals plus gray whale; at least 9 species) and the Ziphiidae (beaked whales; 22 species), have also been problematic to disentangle, with several conflicting internal nodes between studies (Nikaido et al. 2005; Sasaki et al. 2005; Nishida et al. 2007; Dalebout et al. 2008, 2014; Deméré et al. 2008; McGowen et al. 2009; Hassanin et al. 2012). For example, multiple molecular analyses have revealed that the morphologically distinct gray whale (a benthic suction feeder and the sole member of the family Eschrichtiidae) is nested within the engulfment feeding rorquals of Balaenopteridae and relationships at the base of Balaenopteroidea have varied between studies (Nikaido et al. 2005; Sasaki et al. 2005; Deméré et al. 2008; McGowen et al. 2009; Steeman et al. 2009; Hassanin et al. 2012; Árnason et al. 2018). In addition, most data gathered for Ziphiidae, especially the genus *Mesoplodon* (i.e., mitochondrial [mt] genes, 2 nuclear loci), have not robustly resolved species-level relationships (e.g., Dalebout et al. 2008, 2014). There is a pressing need for a good understanding of cetacean systematics, especially in light of their status as highly protected species; smaller cetaceans, in particular, are under increasing threat, as evidenced by the recent extinction of the Chinese river dolphin (*Lipotes vexillifer*; Turvey et al. 2007) and the rapidly declining population of the vaquita porpoise, which may have $<$30 individuals left in the wild (Thomas et al. 2017).

The release of several cetacean genomes and transcriptomes in recent years has made it possible to detail the molecular differences between species, as well as identify variable regions or sites for use in population-level and phylogenetic studies (Gui et al. 2013; Yim et al. 2013; Zhou et al. 2013; Foote et al. 2015; Keane et al. 2015; Tsagkogeorga et al. 2015; Cammen et al. 2016; Warren et al. 2017; Árnason et al. 2018; Zhou et al. 2018). In addition, new advances in state-of-the-art target sequence capture approaches underpinned by short-read high-throughput sequencing technologies means that huge volumes of genetic data (e.g., thousands of genetic markers per sample) are now obtainable from small amounts of starting material at lower cost (Mamanova et al. 2010; Gasc et al. 2016). Such approaches offer unprecedented opportunities for studying the genomes of non-model organisms such as cetaceans and developing methods that can be used by researchers for a diverse array of non-model systems. Target sequence capture shows especially great prospects in phylogenomic studies to investigate the generation of multiple loci for large-scale systematic studies, and utilizing target capture of exons to sequence large numbers of loci has led to increased resolution of vertebrate clades both deep and shallow (McCormack et al. 2013; Bragg et al. 2015; Portik et al. 2016; Schott et al. 2017). However, with the increase in genomic data, reconstructing divergence dates using standard approaches is computationally intensive, and some researchers have called for the use of reduced data sets using clocklike genes (Smith et al. 2018).

To resolve uncertain relationships among cetacean lineages, we generated new sequence data for 3191 protein-coding genes in 68 species of cetaceans, two hippopotamids and three ruminants. By supplementing these data with available sequences from 18 taxa (11 cetacean, 7 outgroup) obtained from a combination of published genomes, transcriptomes and other data sets, our final alignments spanned 100 individuals from 77 cetacean and 12 outgroup species. We used more than 6.5 million bp of aligned sequence from 38,167 exons of 3191 genes to construct a large-scale well-supported species tree of Cetacea using both concatenated and coalescence methods. Every node was well-resolved, including those within Ziphiidae and the problematic Delphinidae (oceanic dolphins). Our results resolve a long debate over the contentious relationships among species within the subfamily Delphininae, which includes some of the most recognizable cetaceans, such as common dolphins (*Delphinus*) and bottlenose dolphins (*Tursiops*). Importantly, our large data set also allowed us to unravel the pattern of molecular rate variation in cetaceans, and thus obtain a precise species-level timetree of cetacean divergences using our complete data set.

## Materials and Methods

### Sample Description, DNA Extraction, and Library Construction

We obtained tissue or DNA from national repositories for 68 species (77 total individuals) of cetaceans, two species of hippopotamuses, and three species of ruminants (Appendix Table A1). These DNAs were extracted using Qiagen DNeasy extraction kits (Qiagen UK Ltd., Manchester, UK). DNA quality was then evaluated using the Agilent Tape Station 2200, and $\sim $100–200 ng per sample was sheared using a Covaris focused ultrasonicator to achieve $\sim $200 bp fragments. Some degraded samples of $< $100 ng were not sheared due to their already fragmentary nature. After shearing, fragment size, quantity, and quality of the DNA were then determined using the Agilent 2100 Bioanalyzer. Illumina libraries were constructed for each sample using the NEBNext Ultra and Ultra II DNA Library Prep Kits with NEBNext Multiplex Oligos (Dual Index Primers Set 1; New England Biolabs, Ipswich, MA, USA) and the standard protocol provided. The Bioanalyzer was then used to assess success of library construction before further amplification using 6–12 cycles.

### Design of Biotinylated RNA Baits

A list of 1:1 orthologous protein-coding genes for the *Tursiops truncatus* genome version turTru1 (as compared with protein-coding genes from *Homo sapiens* and other available laurasiatherians) was compiled using Ensembl v. 75. We included genes belonging to specific gene ontology (GO) categories based on genes of interest and added these to a larger subset of randomly selected genes. Our target loci covered a range of GO categories ranging from “regulation of centrosome cycle” to “lung development”. Official HGNC gene names were used to search the coding sequence (CDS) databases of two delphinid genomes, *Tursiops truncatus* (version Ttru_1.4) and *Orcinus orca* (version Oorc_1.1) on NCBI GenBank (Foote et al. 2015). The longest CDS for each gene, whether *Tursiops* or *Orcinus*, was downloaded. For some sequences, no delphinid sequence was available, and another cetacean CDS was used (*Lipotes vexillifer*, *Physeter macrocephalus*, *Balaenoptera acutorostrata*; Zhou et al. 2013; Yim et al. 2013; Warren et al. 2017). This resulted in 10,271 individual CDS sequences with a total of 18,386,718 bp.

Biotinylated RNA baits (MYbaits) of 100 nucleotides in length were designed by MYcroarray (Ann Arbor, MI, USA; now Arbor Biosciences) using these 10,271 individual CDS sequences. Baits were evaluated via a MYcroarray in-house algorithm, and those with potential to cause cross-hybridization to multiple targets (based on the *T. truncatus* genome [version Ttru_1.4] as a reference) were filtered using a relaxed “4” setting. We then initiated a pilot study of four cetaceans (*Mesoplodon bidens*, *Lagenodelphis hosei*, *Caperea marginata*, *Stenella coeruleoalba*), and the pygmy hippopotamus (*Choeropsis liberiensis*), to determine the success of target sequence capture before proceeding further.

After target sequence capture of these five species using the same protocols described below, we reduced the number of included genes to 3256 based on the success of capture of at least two species for a majority of exons of a particular gene. We constructed a new round of baits for these sequences using the same parameters. We then used these baits to capture DNA sequences from all 77 individuals, representing 68 species cetacean, both species of hippopotamuses, and three species of ruminants (Appendix Table A1).

### Target Sequence Capture and Sequencing

Target sequence capture was performed following the protocol contained in MYBaits Manual version 3.0, in which biotinylated RNA baits were hybridized to individual sample libraries for $\sim $20–24 h. Captured DNA was recovered using Streptavidin C1 magnetic beads (MyOne) and washed to remove any unhybridized fragments. Then all captured DNA was amplified and individual samples were pooled into two batches for sequencing. Each batch was paired-end sequenced by the “Bart’s and the London Genome Centre” of Queen Mary, University of London using the Illumina NextSeq 500 platform with the high output mode and a read length of 150 bp. All raw reads were deposited in the Sequence Read Archive (SRA) of NCBI, BioProject PRJNA575269.

### Assembly of Reads and Identification of Contigs

We assessed the quality of raw reads using FastQC version 0.11.5 (Babraham Bioinformatics), and raw reads were cleaned by removing adaptors and low-quality bases using Trimmomatic 0.36 (Bolger et al. 2014). A total number of reads for each sample are shown in Supplementary Table S1 available on Dryad at https://doi.org/10.5061/dryad.jq40b0f. Trimmed reads for each individual sample were *de novo* assembled separately using Trinity v2.2.0 with default settings (Grabherr et al. 2011). To identify each Trinity contig, we then conducted reciprocal blast searches (blastn; $E$-value cutoff of 10$^{{\rm t}-6}$; retained only top blast hit) of each Trinity assembly using FASTA files with all exons drawn from the *O. orca* and *T. truncatus* genomes. Per species counts of contigs with a reciprocal blast hit are shown in Supplementary Table S1 available on Dryad. In addition, we also conducted reciprocal blast searches with FASTA files containing CDSs from existing cetacean genomes, partial genomes, or transcriptomes including *Balaenoptera acutorostrata*, *B. physalus*, *Megaptera novaeangliae*, *Balaena mysticetus*, *Physeter macrocephalus*, *Neophocaena phocaenoides*, *Lipotes vexillifer* (Zhou et al. 2013; Yim et al. 2013; Keane et al. 2015; Tsagkogeorga et al. 2015; Warren et al. 2017), as well as outgroup genomes from *Bos taurus*, *Ovis aries*, *Panthalops hodgsonii, Sus scrofa, Vicugna pacos, Camelus bactrianus*, and *Equus caballus* (Lindblad-Toh et al. 2011; Bactrian Camels et al. 2012; Groenen et al. 2012; Ge et al. 2013; Jiang et al. 2014). All contigs were then trimmed to the length of the desired exon. We then kept all exons (38,832) present in our original baits for further downstream analyses.

### Alignments

Each individual exon was aligned separately using mafft version 7 (Katoh and Standley 2013) for a total of 38,832 exon alignments. These were then concatenated into complete gene alignments. To assess the efficacy of this process, alignments were then translated into amino acids to identify potential stop codons. Alignments with stop codons were examined by eye. In some cases, insertions at the end of exon boundaries were introduced from blastn, and these were removed. In other cases, exons were missing from the original annotations of the *O. orca* and *T. truncatus* genomes; these exons were then introduced in order for the whole alignment to remain in the correct reading frame. In addition, we removed 65 genes (665 exons) from the overall data set if the gene was difficult to align or difficult to differentiate sequences from closely related paralogues. For the remaining 3191 genes, presence of premature stop codons and/or indels that were not multiples of three nucleotides were taken as potential evidence for the presence of a pseudogene and noted for further analysis.

We also added isolated sequences from NCBI GenBank for *Platanista gangetica* (South Asian river dolphin) and *Balaenoptera omurai* (Omura’s whale), two species for which we did not have capture data and for which a whole genome is not available. This consisted of a total of 72 sequences for *P. gangetica* (57,770 bp) and 67 for *B. omurai* (57,686 bp). See Supplementary Table S2 available on Dryad for a list with accession numbers of these sequences and their publications.

### Phylogenomic Analyses

We created two concatenated alignments, both with a total of 3191 genes (38,167 exons) and 6,527,596 bp: Dataset A and Dataset B. Dataset A contained sequences from *P. gangetica* and *B. omurai*, whereas Dataset B excluded these sequences. For both data sets, we conducted three concatenated maximum likelihood analyses using RAxML v8.2 (Stamatakis 2014): (i) unpartitioned, (ii) 3191 partitions, one for each gene, and (iii) a partition scheme of 1573 partitions selected using PartitionFinder v2.1.1 (Lanfear et al. 2016). We performed each analysis using the GTRCAT model for every partition. Each analysis used default parameters in RAxML and support scores were generated using the rapid bootstrapping option with at least 1000 replicates. To confirm our findings using an alternative method, a Bayesian analysis of Dataset A was implemented in ExaBayes using default parameters and a GTR+G model of evolution (Aberer et al. 2014). Two unpartitioned analyses of Dataset A were conducted for 1,000,000 generations with two coupled chains instituted for each analysis and trees sampled every 500 generations. The initial 25% of runs were discarded as burn-in. Results of the Bayesian analyses were examined in Tracer v1.7 (Rambaut et al. 2018) to evaluate whether parameters, node ages, and likelihood values had converged. All RAxML and ExaBayes runs were implemented using the CIPRES Science Gateway v3.3 (Miller et al. 2010).

We also implemented a species tree analysis, which takes into account the potential discordance between individual gene trees and the underlying species tree due to incomplete lineage sorting. We first generated individual maximum likelihood gene trees in RAxML v8.2 for each of the 3191 genes using a GTRCAT model. Due to the comparatively small number of sequences present for *P. gangetica* and *B. omurai*, we excluded them from all gene tree analyses. We used ASTRAL-III v5.6.1 to generate a species tree using a multi-species coalescent model (Mirarab and Warnow 2015; Zhang et al. 2018). We used as an input the best-scoring ML trees from each separate 3191 RAxML gene tree analysis. Individuals from the same species were constrained as monophyletic.

### Divergence Dating Analysis

For our divergence dating analyses, we reduced the subset of genes and taxa used. We used only genes with no evidence of pseudogenization (internal stop codons, frameshift mutations), reducing the number of loci included to 3096. In cases where more than one representative of a particular species was present, we retained the more complete individual; however, two representatives were retained for *Delphinus delphis*, the *delphis* short-beaked form, and one of the *bairdii* long-beaked forms (108471). In addition, we excluded species missing $>$50% of their exons (i.e., *Hyperoodon planifrons*, *Phocoenoides dalli*, *Berardius arnuxii*, *P. gangetica*, *B. omurai*). Operational taxonomic units (OTUs) were pruned from the topology generated from our concatenated analyses (all RAxML and ExaBayes analyses resulted in the same topology), and the resulting fixed tree with 85 taxa was used as an input for downstream analyses.

Due to the computational difficulties of analyzing each gene as a separate partition, we followed the procedure outlined in dos Reis et al. (2012) and grouped genes with similar relative rates of divergence. The “baseml” package in PAML v4.9h (Yang 2007) was used to generate pairwise distance matrices for each of 3096 genes using the HKY85 model of molecular evolution (Hasegawa et al. 1985). Pairwise distances between *O. orca* (an odontocete) and *B. acutorostrata* (a mysticete) were compiled for each gene; however, in some cases, *B. acutorostrata* was not present and another mysticete was used. Using pairwise distances, this data set was divided into 3 and 10 partitions of 1032 and approximately 309 genes each, respectively, representing partitions ranging from slower to faster rates of divergence. The three-partition data set was further split into “first and second” and “third” codon positions (1st/2nd and 3rd CPs) for a total of six partitions. In total, we analyzed a 3-partition scheme separated by rate of divergence, a 6-partition scheme by rate of divergence and codon position, and a 10-partition scheme by rate of divergence.

Divergence dating analyses were conducted using the software MCMCTree v4.9h, part of the PAML package (Yang 2007). MCMCTree implements approximate likelihood calculation allowing Bayesian divergence time inference of phylogenomic data sets (dos Reis and Yang 2011; dos Reis et al. 2012). Marginal likelihoods for relaxed-clock models were calculated using the stepping-stones method (Xie et al. 2011) as implemented in the mcmc3r R package (dos Reis et al. 2018). The marginal likelihoods were then used to calculate posterior probabilities (PP) for the strict, autocorrelated and independent rate models (AR and IR, respectively). The approximate likelihood method cannot be used for marginal likelihood calculation (dos Reis et al. 2018) and thus the computationally expensive exact method must be used. Therefore, to decide the best-fitting clock model, we carried out Bayesian model selection on smaller subsets of the data suitable for exact likelihood calculation: 1 randomly selected locus for subsets of 20, 40, and all 85 species; 5 randomly selected loci for a subset of 20 species; and 20 randomly selected loci for a subset of both 20 and 40 species. As in dos Reis et al. (2018), for analyses which used less than 85 species, we chose taxa from representative clades to reflect the true diversity of rate variation across taxa. Note that the sampling of genes was random and, based on inference theory, we had no reason to expect any biases in model selection.

Test runs of the program were carried out to ensure the convergence of the Markov Chain Monte Carlo (MCMC) chains and that enough likelihood samples had been collected for Bayes factors calculation. The birth–death process with $\lambda $ =$\mu $ = 1 (birth and death rates) and *$\rho $* = 0.1 (fraction of species sampled) was used to construct the prior on node ages. These parameters lead to an approximately uniform density on node ages (Yang and Rannala 2006). At this stage, we did not want to estimate divergence times but simply select the most appropriate clock model given the data, thus the root age was fixed to 1. In MCMCTree, this may be done by using a narrow uniform distribution between 0.999 and 1.001. No other fossil calibrations were used at this step. We used the HKY85+$\Gamma _{5}$ substitution model (Hasegawa et al. 1985; HKY model accommodating among site rate heterogeneity using a gamma distribution with five categories), and a diffuse gamma-Dirichlet prior (dos Reis et al. 2014) for both the molecular rate, $\Gamma $(2, 20), and the diffusion rate $\sigma^{2}$, $\Gamma $(2, 2). In all cases, the autocorrelated-rates model was determined to be the most appropriate based on the subsets of data (Table 1).

**Table 1.** Bayesian selection of the relaxed-clock model

Data	Model	log mL $\pm $ S.E	Pr
1g, 20s	AR	$-4176.387 \pm 0.026 $	0.993
	IR	$-4181.350 \pm 0.019 $	0.007
	STR	$-4194.797 \pm 0.016 $	0
1g, 40s	AR	$-4957.$026 $\pm $ 0.050	1
	IR	$-4973.$258 $\pm $ 0.040	0
	STR	$-5010.$601 $\pm $ 0.047	0
1g, 85s	AR	$-6239.$864 $\pm $ 0.059	1
	IR	$-6258.$492 $\pm $ 0.069	0
	STR	$-6322.$348 $\pm $ 0.043	0
5g, 20s	AR	$-22529.$810 $\pm $ 0.035	0.999
	IR	$-22536.$580 $\pm $ 0.030	0.001
	STR	$-22555.$840 $\pm $ 0.022	0
20g, 20s	AR	$-94729.$470 $\pm $ 0.043	0.998
	IR	$-94738.$010 $\pm $ 0.058	0.002
	STR	$-94838.$540 $\pm $ 0.038	0
20g, 40s	AR	$-110512.$300 $\pm $ 0.181	1
	IR	$-110530.$800 $\pm $ 0.218	0
	STR	$-110668.$500 $\pm $ 0.130	0

*Notes:* Data list each treatment with the number of genes (g) and species (s) for each alignment. Models tested include AR, autocorrelated rates; IR, independent rates; STR, strict clock. Log mL + SE is the log-marginal likelihood for the model with standard error for the log-likelihood estimate. Pr is the posterior model probability (assuming equal prior probabilities for models), calculated as in dos Reis et al. (2018, Appendix 2).

MCMCTree was used to estimate divergence times on the complete data set for the 3-, 6-, and 10-partition schemes using the autocorrelated-rates model as well as the independent rates model for comparison, with both models using approximate likelihood (dos Reis and Yang 2011). All parameters were the same as above, except we used the fossil calibrations in Table 2. MCMC runs were conducted twice for 1 $\times $ 10$^{7}$ iterations with a sampling frequency of 500; the first 50% of each run was discarded as burn-in. Results were examined in Tracer v. 1.7 (Rambaut et al. 2018) to evaluate whether parameters, node ages, and likelihood values had converged. We checked that the estimated sample size (ESS) for each parameter was not smaller than 100 (Nascimento et al. 2017).

**Table 2.** List of calibration dates (minimum and maximum ages), nodes, and rationale for choice of calibration dates used in the MCMCTree divergence dating analysis

Node	Minimum age (Ma)	Minimum rationale	Maximum age (Ma)	Maximum rationale	Citation
Perissodactyla + Cetartiodactyla (Root)	Hard 52.40	*Himalayacetus subathuensis* (oldest crown cetartiodactyl)	Soft 164.6	Maximum age for Laurasiatheria: *Juramaia* (oldest eutherian)	Benton et al. (2015)
Crown Cetartiodactyla	Hard 52.40	*Himalayacetus subathuensis* (oldest crown cetartiodactyl)	Soft 66.00	absence of crown cetartiodactyls	Bajpai and Gingerich (1998); O’Leary and Uhen (1999); Benton et al. (2015)
Cetruminantia	Hard 52.40	*Himalayacetus subathuensis* (oldest crown cetartiodactyl)	Soft 66.00	absence of crown cetartiodactyls	Bajpai and Gingerich (1998); O’Leary and Uhen (1999); Benton et al. (2015)
Whippomorpha	Hard 52.40	*Himalayacetus subathuensis* (oldest crown cetartiodactyl)	Soft 66.00	absence of crown cetartiodactyls	Bajpai and Gingerich (1998); O’Leary and Uhen (1999); Benton et al. (2015)
Crown Bovidae	Hard 16.00	*Pseudotragus seegrabensis* (oldest crown bovid)	Soft 28.00	absence of crown bovids	Bibi (2013); Benton et al. (2015)
Crown Cetacea	Hard 36.40	*Mystacodon selenensis* (oldest crown cetacean)	Soft 52.40	*Himalayacetus subathuensis* (oldest crown cetartiodactyl)	Lambert et al. (2017); Bajpai and Gingerich (1998)
Crown Mysticeti	Hard 25.20	*Mauicetus parki* (oldest crown mysticete)	Soft 36.40	*Mystacodon selenensis* (oldest crown cetacean)	Marx and Fordyce (2015); Lambert et al. (2017)
Crown Ziphiidae	Hard 13.20	*Archaeoziphius microglenoideus* (oldest crown ziphiid)	Soft 23.00	*Notocetus vanbenedeni* (oldest crown synrhinan)	de Muizon (1987); Lambert and Louwye (2006); Geisler et al. (2011)
Phocoenidae + Monodontidae	Hard 7.50	*Salumiphocoena stocktoni* (oldest crown Phocoenidae + Monodontidae)	Soft 19.50	*Kentriodon pernix* (oldest crown delphinidan)	Kellogg (1927); Wilson (1973); Geisler et al. (2011);
Delphinidae exclusive of *L. albirostris*	Hard 8.5	*Eodelphinus kabatensis* (stem *Orcinus*)	Soft 19.50	*Kentriodon pernix* (oldest crown delphinidan)	Kellogg (1927); Murakami et al. (2014)
Delphininae exclusive of *S. guianensis*	Hard 3.98	*Etruridelphis giulii* (oldest crown delphinine)	Soft 8.5	*Eodelphinus kabatensis* (stem *Orcinus*)	Bianucci (2013); Murakami et al. (2014)

With the advent of phylogenomic-scale data sets, computational cost has increased and thus some authors have suggested selecting clocklike genes as a way of reducing data size (Smith et al. 2018). For example, the Python package SortaDate identifies and ranks genes for use in divergence dating analyses based on three criteria: adherence to a molecular clock-like model of divergence, degree of information content, and topological agreement with the species tree (Smith et al. 2018). To compare our results using the complete data set, we conducted analyses using the top 10 genes selected by SortaDate (*ABCA4*, *PTPRZ1*, *TNC*, *COL12A1*, *HYDIN*, *APOB*, *CENPF*, *C2CD3*, *CEP152*, and *LRKK2*). These genes account for a total of 87,864 aligned bp, with individual gene alignments included ranging between 5,202 and 15,381 bp. To directly compare the SortaDate genes with the complete data sets, the 10 genes were ordered from slowest to fastest evolving as above, and we partitioned the data sets into 3, 6, and 10 partitions. For the 3-partition data set, three to four genes each were included in three partitions from slowest to fastest. For the 6-partition data set, these partitions were split into 1st/2nd CPs and 3rd CPs. For the 10-partition data set, each gene was analyzed separately. To assess how uncertainty in time estimates differed between analysis of the whole data set and the 10 SortaDate genes, we used the infinite-sites plot (Rannala and Yang 2007), in which uncertainty in time estimates (measured as the credibility-interval width) is plotted against the posterior mean of node ages. This plot reveals the approximate amount of information content in the molecular data with respect to divergence time estimates (Rannala and Yang 2007; Inoue et al. 2010).

## Results

### Target Sequence Capture

The number of reads recovered per sample ranged from $\sim $4.7 million (*Mesoplodon grayi*) to $\sim $28.9 million (*Stenella attenuata* 38219; Supplementary Table S1 available on Dryad) with an average of $\sim $13 million. Phylogenetic distance from the *Tursiops* and *Orcinus* genomes did not appear to affect the success of sequence capture, as 21.6 million reads were obtained for the ruminant *Gazella arabica*. For each sample, reads were assembled into Trinity contigs numbering from 11,156 (*H. planifrons*) to 575,798 (*Mesoplodon carlhubbsi*) with an average N50 of 310 bp. After reciprocal blasting of the contigs to the *Orcinus* genome using blastn, we recovered between 7428 (*H. planifrons*) and 31,888 exons (*Stenella longirostris* 24923), with an average of 28,324 exons per species (74% recovery of initial exons). Delphinids had a higher average of 30,106 exons (79% recovery). The five noncetaceans ranged from 21,259 to 26,179 exons, with an average of 24,085 exons (63% recovery).

### Phylogenomic Analysis

**Figure 2.**
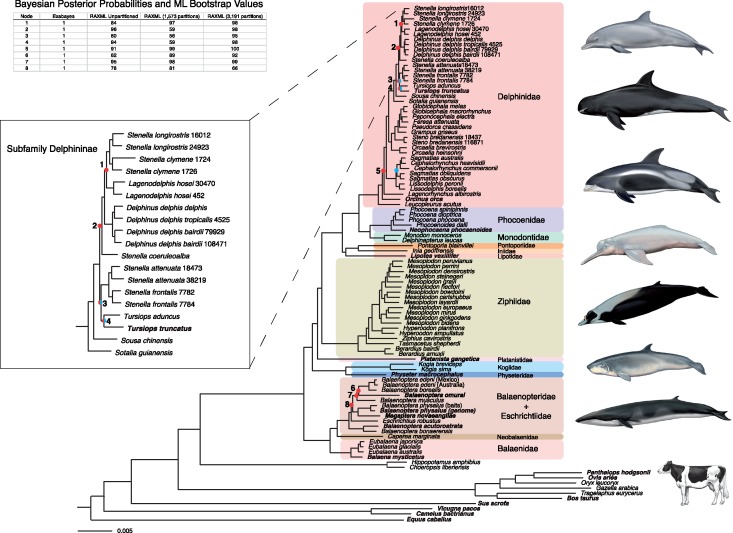
The best concatenated RAxML maximum likelihood tree derived from Dataset A using 3191 partitions of each protein-coding gene and 6,527,596 bp (lnL = $-$24,850,884.943687). The phylogenetic relationships of Delphininae are shown more clearly in the box on the left. All concatenated RAxML and Bayesian analyses using Dataset A retrieved the same topology. All nodes have 100% BS values (RAxML) or 1.0 PP (Exabayes) with the exception of the numbered nodes represented by red dots. These nodes have BS values and PP shown in the table in the upper left. The ASTRAL species tree topology only differed from the concatenated topology at three nodes shown in blue. Taxa in bold are those with data derived from previously existing genomes, transcriptomes, or GenBank sequences. Illustrations are by Carl Buell and represent (top to bottom) *T. truncatus* (common bottlenose dolphin), *Feresa attenuata* (pygmy killer whale), *L. albirostris* (white-beaked dolphin), *Inia geoffrensis* (Amazon river dolphin), *Mesoplodon layardii* (strap-toothed whale), *Kogia sima* (dwarf sperm whale), *B. bonaerensis* (Antarctic minke whale), and *B. taurus* (domestic cow).

The same tree topology was generated with all concatenated analyses of Dataset A using RAxML or ExaBayes, regardless of model or partitioning scheme. In addition, topologies resulting from all analyses of Dataset B agreed with those of Dataset A when *Platanista* and *B. omurai* were pruned. The phylogenomic tree resulting from the RAxML analysis with 3191 separate partitions by gene is shown in Figure 2. Support scores only differed among the separate RAxML analyses at eight nodes (indicated by red dots; Fig. 2), otherwise they showed 100% bootstrap (BS) support (Fig. 2). Of the eight nodes that differed, only four of these had support scores less than 90% BS, two within balaenopterids and two within delphinine dolphins (Fig. 2). Both independent runs using ExaBayes showed evidence of convergence (all ESS values $>$224; Supplementary Fig. S2 available on Dryad) and resulted in a topology with all nodes supported by Bayesian PP of 1.0. All species in which there were 2+ representatives were supported as monophyletic with high support (all BS 100; PP 1.0).

The species tree generated by the coalescence analysis using ASTRAL (Supplementary Fig. S3 available on Dryad) differs from the concatenated analyses at only three nodes, all within Delphinidae (Fig. 1). In addition, all nodes save three have support scores $\ge $0.99 (Supplementary Fig. S3 available on Dryad). The ASTRAL tree places *Lissodelphis* and *Sagmatias obliquidens* + *Sa. obscurus* in a clade to the exclusion of *Cephalorhynchus* + *Sagmatias australis* with posterior probability of 0.8, as well as displacing *T. truncatus* from the clade including *T. aduncus*, *Stenella attenuata* + *frontalis* with high support (Fig. 1 and Supplementary Fig. S3 available on Dryad). Two additional nodes within Delphininae (Nodes 1 and 2; Fig. 2) are supported by the ASTRAL tree but show PP of 0.42 and 0.89, respectively (Supplementary Fig. S3 available on Dryad), agreeing with the varying support among the same nodes in the concatenated analyses. The ASTRAL species tree has a final normalized quartet score of 0.869, representing the proportion of quartets for individual gene trees that are satisfied by the species tree.

All analyses supported the monophyly of Cetruminantia (Cetacea + Hippopotamidae + Ruminantia), Ruminantia, Whippomorpha (Cetacea + Hippopotamidae), Cetacea, Odontoceti, Mysticeti, Synrhina, Delphinida, Physeteroidea, Inioidea, Delphinoidea, *Lipotes* + Inioidea, Phocoenidae + Monodontidae (Monodontoidae *sensu*Geisler et al. 2011), and all recognized cetacean families with the exception of Balaenopteridae. Several genera were well-supported as polyphyletic, including *Balaenoptera*, *Sagmatias* (*sensu*Leduc et al. 1999; Vollmer et al. 2019), *Cephalorhynchus*, and *Stenella*. Dataset A clearly supports *P. gangetica*, the South Asian river dolphin, as a separate lineage from the other “river” dolphins (*Lipotes*, *Inia*, *Pontoporia*) and also supports its exclusion from the clade Ziphiidae + Delphinida.

### Divergence Dating

**Figure 3.**
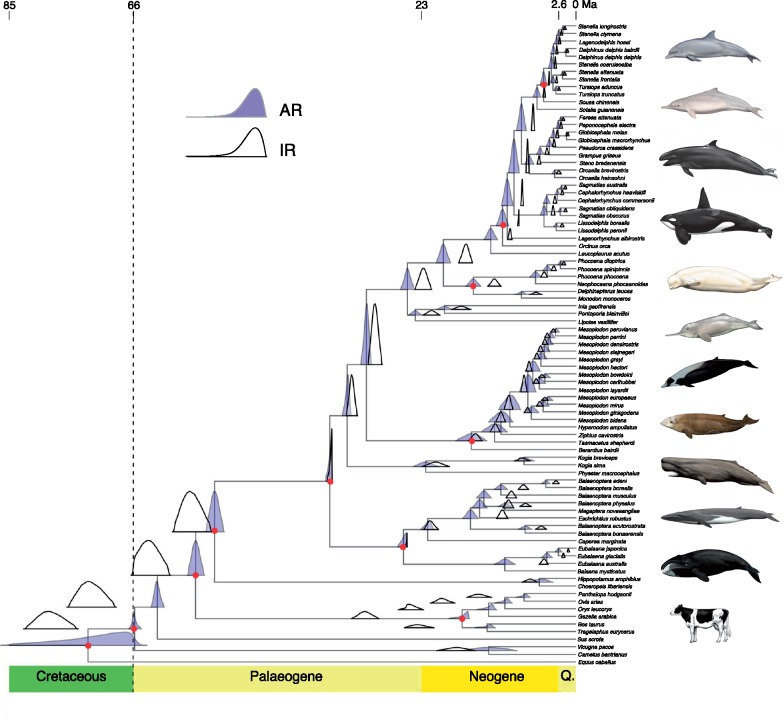
Timetree of Cetacea analyzed in the MCMCTree package of PAML 4.9h using six partitions and approximate likelihood (Yang 2007). A time scale in Ma (millions of years) is shown above the tree, with geologic periods labeled below the tree for reference (Q = Quaternary). Above each node the posterior distributions of the AR model (purple) and IR model (white) are shown. Raw numbers for the mean and 95% confidence intervals for each node and each model are shown in Supplementary Table S3 available on Dryad. Red circles at each node represent calibration points listed in Table 2. Illustrations are by Carl Buell and represent (top to bottom) *T. truncatus* (common bottlenose dolphin), *Sousa chinensis* (Indo-Pacific humpback dolphin), *F. attenuata* (pygmy killer whale), *O. orca* (killer whale), *Delphinapterus leucas* (beluga), *L. vexillifer* (Yangtze river dolphin), *Mesoplodon layardii* (strap-toothed whale), *Ziphius cavirostris* (Cuvier’s beaked whale), *P. macrocephalus* (sperm whale), *B. physalus* (fin whale), *B. mysticetus* (bowhead whale), and *B. taurus* (domestic cow). This tree with posterior node estimates was generated with the mcmc.tree.plot function from the R package MCMCtreeR (Puttick 2019).

Table 1 shows the results of the Bayesian model selection analysis, which was used to determine the best-fitting model in subsequent MCMCTree analyses. For all sampled alignments, the AR model had the highest posterior probability ($\sim $1.0 in all cases) and was interpreted as the best-fitting model to our data. All comparable MCMCTree runs for the total data set using each partition scheme and model showed evidence of convergence with an ESS for each parameter $>$200 (Supplementary Figs. S4–S9 available on Dryad). The timetree of Cetacea obtained using the 6-partition AR model is shown in Figure 3 with the posterior probability distributions of both AR and IR models shown above each node. For comparison, we ran MCMCTree using a 3-partition and 10-partition model, and the timetrees for both are shown in Supplementary Figures S10 and S11 available on Dryad with posterior probability distributions of both AR and IR models above each node. Precise dates (mean and 95% CI) for both AR and IR models for the 3-, 6-, and 10-partitions of the complete data set are listed in Supplementary Table S3 available on Dryad using the numbers for each node labeled in Supplementary Figure S12 available on Dryad. Divergence dates for distinct nodes are similar among the three-partition schemes for each model (AR and IR), although within Delphinida divergence times generally decrease slightly with the increase in partitions for the AR model (Supplementary Table S3 available on Dryad). For example, the mean date of divergence within Delphinida decreases by an average of 1.25 Ma with the increase from 3-partitions to 10-partitions (Supplementary Table S3 available on Dryad).

**Figure 4.**
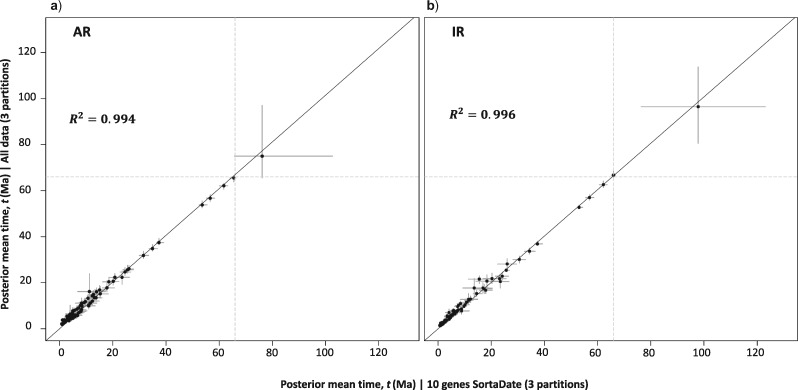
Scatterplot of the estimated posterior mean times (and 95% confidence intervals) for the six-partition scheme of both AR (a) and IR (b) models for the SortaDate data set ($x$-axis) against all data ($y$-axis).

**Figure 5.**
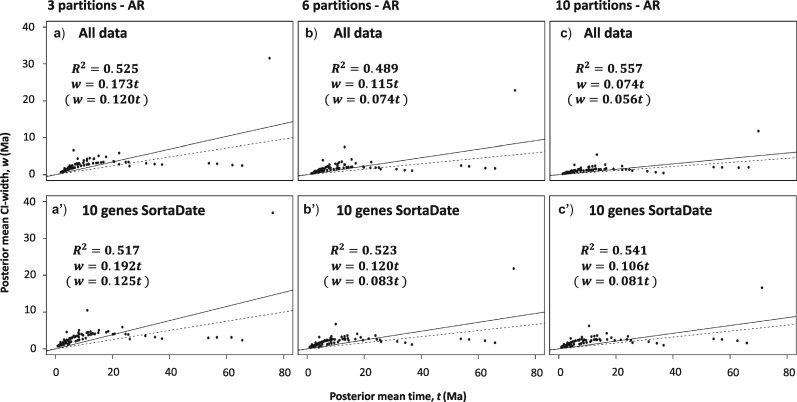
Infinite-sites plots showing the estimated posterior mean times in Ma ($x$-axis) plotted against the estimated posterior confidence interval (CI) widths in Ma ($y$-axis) for the AR model for both data sets (all data, 10 genes SortaDate) using the three different partition schemes, three partitions (a, a’), six partitions (b, b’), and 10 partitions (c, c’). The solid line represents the regression line including the root and the dotted line represents the regression line excluding the root. $R^{2}$ is the coefficient of determination for each comparison, whereas below each are the equations of the regression lines with and without the root.

Differences in posterior mean times do not differ drastically between using 10 genes (SortaDate) and using the entire data set (Fig. 4, Supplementary Figs. S13 and S14, Table S3 available on Dryad); however, variances are generally larger when using the reduced data set (Fig. 5, Supplementary Fig. S15, Table S3 available on Dryad). For example, when analyzing the 10 SortaDate genes as three partitions under the AR model (Fig. 5a’), the slope of the regression line in the infinite-sites plot is 0.192, implying that for every 1 Ma of divergence, 0.192 Ma of uncertainty are added to the 95% CI. When we include all the data in three partitions (Fig. 5a), the regression slope falls to 0.173, and it falls further to 0.115 and 0.074 when analyzed as 6 and 10 partitions, respectively (Fig. 5b,c). Thus, the analysis using the whole data set provides time estimates with the narrowest credibility intervals. The same trend is seen with the exclusion of the root (Fig. 5), as well as using the IR model (Supplementary Fig. S15 available on Dryad). We note that in none of the plots do points form a straight line. This indicates that uncertainty in time estimates are due both to limited data as well as uncertainties in the fossil calibrations (Rannala and Yang 2007).

Outside of Cetacea (and exclusive of the root), the mean age of nodes using the 6-partition model decreased using the AR model with respect to IR by an average of 8.29 Ma, with the mean age of Bovidae shifting by 14.47 Ma (Fig. 3; Supplementary Table S3 available on Dryad). Within Cetacea, nodes increased using the AR model with respect to the IR model by an average of 1.08 Ma, although most nodes within Ziphiidae, as well as *Kogia* decreased by $>$0.61 (Fig. 3; Supplementary Table S3 available on Dryad). At least eight nodes within Cetacea increased by over 3 Ma when using the AR as compared with the IR model, including Crown Balaenidae (10.61 Ma vs. 4.79 Ma), Delphinoidea (19.78 Ma vs. 16.44 Ma), and Balaenopteroidea (15.74 Ma vs. 10.99 Ma). Nodes within mysticetes differed widely between analyses by an average of 3.07 Ma. Results are comparable when using the other partitioning schemes (Supplementary Table S3 available on Dryad).

Using the 6-partition AR model, we obtained a mean age for Whippomorpha ($\bar{x}= 53.92$ Ma), less than a million years before the earliest stem cetacean, *Himalayacetus subathuensis* (Bajpai and Gingerich 1998; Supplementary Table S3 available on Dryad). The age of Crown Cetacea is much more recent ($\bar{x}=36.72$ Ma), which is less than half a million years older than the oldest-known crown cetacean fossil, the stem mysticete *Mystacodon selenensis* from the Late Eocene of Peru (Lambert et al. 2017). The diversification of Crown Odontoceti began before the end of the Eocene ($\bar{x}= 34.13$ Ma), whereas the emergence of Crown Mysticeti ($\bar{x}= 25.73$ Ma) is more than 8 myr more recent, firmly within the Oligocene. All lineages leading to modern cetacean families were present by the Middle Miocene. Balaenopteroidea, Ziphiidae, Monodontidae + Phocoenidae, and Delphinidae began to diversify in the Early-to-Middle Miocene, with diversification of the speciose genus *Mesoplodon* and the delphinid subfamilies beginning in the Late Miocene.

## Discussion

The evolution of the cetaceans, from their early transition to an aquatic niche to their rapid diversification, has been the subject of numerous studies, yet several aspects of their systematics have remained unresolved. Using a targeted sequence approach, in which we constructed RNA baits for exons based on the *O. orca* and *T. truncatus* genomes, we were able to obtain sequences for an average of 74% of target exons from most of the cetacean species as well as their closest relatives. Our final data set of 38,167 exons contains the first ever large-scale genomic data for at least 58 cetacean species, as well as for the pygmy hippopotamus, and allowed us to produce a fully resolved, time-calibrated tree that was able to elucidate with confidence several problematic relationships.

### Divergence Dating

Several studies have indicated that molecular rates within cetaceans, especially within mysticetes, are much slower than those in other mammals (Bininda-Emonds 2007; Jackson et al. 2009; Dornburg et al. 2012). However, estimates of rates and times may be substantially affected by the relaxed-clock model used (e.g. dos Reis et al. 2018), and thus it is important to select the most appropriate clock model. For example, Dornburg et al. (2012) found that uncorrelated rate models perform poorly compared with local clocks when there is significant rate variation between lineages. An appealing property of the AR model implemented in MCMCTree is that it allows for local clocks in more closely related species whereas allowing for rates to vary more widely in deeper nodes, a property that appears biologically realistic (dos Reis et al. 2018). In this study, we find that like dos Reis et al. (2018), the AR model was preferred based on analysis of a subset of the data (Table 1), and estimates of times are substantially affected depending on the rate model used (Fig. 3; Supplementary Table S3 available on Dryad). However, unlike primate estimates in dos Reis et al. (2018), the AR divergence estimates produced more recent estimates for deeper nodes than the IR model, whereas tending to skew older within cetaceans (Fig. 3). For some dates along the trunk of the cetacean tree (i.e., Delphinidae), the AR analysis obtained slightly older dates than previous analyses which have used divergence dating analyses with uncorrelated rates (McGowen et al. 2009; Slater et al. 2010; Zhou et al. 2011) or penalized likelihood (Steeman et al. 2009); although all used substantially less data.

The approximate likelihood method (dos Reis and Yang 2011) has a disadvantage in that the marginal likelihood cannot be directly computed. Only by carrying out the MCMC analysis using the exact likelihood can we then estimate the marginal likelihood, but this is not computationally feasible with very large data sets. We have attempted to overcome this computational limitation by using Bayesian model selection on various subsets of our data (Table 1). Our results show that for all subsets analyzed, the preferred model is AR, although more powerful computational approaches are needed so we can validate these results with the whole data set. Nevertheless, our analyses based on the stepping stone method seem to indicate that the AR model better explains the rate variation among cetaceans and outgroups.

Computational difficulties in analyzing large-scale genomic data sets have led some to suggest using a reduced set of loci (Smith et al. 2018). However, Rannala and Yang (2007) show that uncertainties due to finite-data sampling and the relaxed-clock model approach zero asymptotically as both the number of loci (i.e., the number of partitions) and the number of sites in each locus approach infinity. Rannala and Yang (2007) also show that uncertainties due to fossil calibrations cannot be eliminated. Thus, to reduce uncertainty in time estimates, they recommend analysis of large data sets. Rannala and Yang (2007) and Inoue et al. (2010) suggest using the infinite-sites plot to assess whether uncertainty in a Bayesian clock-dating analysis is dominated by the fossil calibrations or by errors due to the finite-data samples. In the infinite-sites plot, points asymptotically approach a straight line as the number of partitions and number of sites in the analysis is increased. For some large data sets, data points should approximate a straight line, indicating that any uncertainties are due to fossil calibrations (e.g. felid data in Inoue et al. [2010]). In such cases, including further molecular data in the analysis will not improve the time estimates, as the sampling errors due to finite data are zero.

We note that in the approximate likelihood method, which we used here to estimate all divergence times, computational time depends on the size of the Hessian matrix used in the approximation, which in turn depends on the number of species analyzed (dos Reis and Yang 2011), but not on the number of sites in an alignment. Thus, MCMC sampling of 100 species takes approximately the same time whether we analyze 10$^{3}$, 10$^{6}$, or 10$^{9}$ sites. This would not have been the case under exact likelihood computation where computational time is proportional to the number of site-patterns in the alignment, and under which genome-scale data cannot be analyzed. Given that analyzing the whole data set produces estimates with considerably less uncertainty than those obtained using the 10 SortaDate genes (Fig. 5), we suggest that the whole data estimates should be preferred in our cetacean analysis.

### Relationships among Major Cetartiodactylan Lineages

We obtained 100% resolution for the relationships of the five major lineages of Cetartiodactyla, with Tylopoda (*Vicugna*, *Camelus*) as the most basal lineage, followed by Suiformes (*Sus*), Ruminantia (*Bos*, *Tragelaphus*, *Gazella*, *Oryx*, *Ovis, Panthalops*), Ancodonta (*Hippopotamus*, *Choeropsis*), and Cetacea. Phylogenomic analysis unequivocally supported a monophyletic clade which included both hippopotamuses and cetaceans (Whippomorpha) to the exclusion of other cetartiodactyls, with a mean age of 53.92 Ma (Fig. 3). The mean age of Crown Cetacea ($\bar{x}= 36.72$ Ma; Fig. 3) agrees with some previous analyses (McGowen et al. 2009; Steeman et al. 2009; Slater et al. 2010), but is slightly older than some other divergence dating analyses of the group (Nikaido et al. 2001; Sasaki et al. 2006; Xiong et al. 2009; Meredith et al. 2011; Hassanin et al. 2012; although see Marx and Fordyce 2015). This is despite using the recently described stem mysticete *M. selenensis* as a calibration for Crown Cetacea (Lambert et al. 2017), the age of which (36.4 Ma) is estimated to be more than 2 Ma older than *Llanocetus denticrenatus* (34.2 Ma), a stem mysticete previously identified as the oldest crown cetacean and used as a calibration point in numerous divergence dating analyses of cetaceans.

### Phylogeny and Evolution of Mysticeti

The age of Crown Mysticeti ($\bar{x} = 25.75$ Ma; 95% CI: 25.22–26.72 Ma) is younger than most estimates including those using mitochondrial genomes and low-coverage genomes of mysticetes (Sasaki et al. 2006; McGowen et al. 2009; Steeman et al. 2009; Árnason et al. 2018). However, many of these analyses used OU 22244 (previously identified as an archaic right whale $\sim $28 Ma) as a constraint for Crown Mysticeti. Marx and Fordyce (2015) identified OU 22244 as falling outside of Crown Mysticeti, and *Mauicetus parki* (the calibration used here; Table 1) as the earliest crown mysticete.

Within baleen whales, our results demonstrate unequivocal support for the monophyly of Balaenidae ($\bar{x}= 10.61$ Ma), as well as for the clade of Neobalaenidae plus Balaenopteroidea (Plicogulae *sensu*Geisler et al. 2011; $\bar{x}= 22.11$ Ma), despite some morphological analyses which support a relationship between Neobalaenidae and Balaenidae (Bouetel and de Muizon 2006; Bisconti 2007, 2014; Steeman 2007; Churchill et al. 2011; El Adli et al. 2014). In agreement with results presented here, most molecular and some morphological analyses support Plicogulae (Árnason and Gullberg 1994; Rychel et al. 2004; Sasaki et al. 2005, 2006; Agnarsson and May-Collado 2008; Deméré et al. 2008; Steeman et al. 2009; McGowen 2011; Hassanin et al. 2012; Fordyce and Marx 2013; Marx and Fordyce 2015). The North Pacific and North Atlantic right whales (*Eubalaena japonica* and *Eubalaena glacialis*, respectively) form an unequivocally well-supported clade (Fig. 2), which conflicts with some weakly supported mtDNA and ncDNA analyses that placed *E. japonica* in a clade with *Eubalaena australis* to the exclusion of *E. glacialis* (Rosenbaum et al. 2000; Gaines et al. 2005). *Eubalaena glacialis* was split into two species (*E. glacialis* and *E. japonica*) partly based on the assumption that *E. japonica* was more closely related to *E. australis* (Rosenbaum et al. 2000). The status of species within *Eubalaena* may have to be reevaluated in light of these results, but the mean age of species events within the genus ($\bar{x}= 4.35$ and $\bar{x}= 2.62$ Ma; Supplementary Table S3 available on Dryad) intimate that the status of *E. japonica* and *E. glacialis* as separate species is warranted.

Our analyses show that the gray whale (*Eschrichtius robustus*; Eschrichtiidae) is firmly nested within Balaenopteridae, in agreement with previous studies based on fewer phylogenetic markers (Árnason and Gullberg 1994; Rychel et al. 2004; Sasaki et al. 2005, 2006; Nishida et al. 2007; Deméré et al. 2008; Agnarsson and May-Collado 2008; Steeman et al. 2009; McGowen 2011; Hassanin et al. 2012). In addition, we find that the genus *Balaenoptera* is polyphyletic with both *Eschrichtius* and *Megaptera* nested within the genus. We show high support for minke whales (*B. acutorostrata* + *Balaenoptera bonaerensis*) diverging earliest within the Balaenopteroidea, followed by *E. robustus* (although BS scores of ML analyses range from 64 to 78). According to our analyses, the radiation of Balaenopteroidea began $\sim $15.74 Ma, and divergence dates within this group are slightly older than some recent analyses (McGowen et al. 2009; Árnason et al. 2018). Indeed divergence dates within Balaenopteroidea vary greatly between our AR and IR analyses (Fig. 3), with the IR analyses showing more recent dates of up to 4.75 Ma.

Árnason et al. (2018) sequenced new low-coverage genomes from six mysticete species and discovered a similar arrangement of balaenopteroid species based on coalescence analyses of trees derived from $>$30,000 20-kb genomic segments, with the exception that the gray whale is sister to *M. novaeangliae* + *B. physalus*. The branch supporting this relationship is incredibly short, and support for conflicting trees is high; their analysis of quartet scores showed that no arrangement between the *Balaenoptera musculus* group, *M. novaeangliae* + *B. physalus*, and *E. robustus* could be significantly rejected. These results were interpreted as implying that large-scale hybridization played a part early in balaenopteroid evolution. Analysis of retrotransposon insertion events using the same genomes reveals a similar pattern (Lammers et al. 2019). Whatever the cause, Árnason et al. (2018) and the results presented here both agree that a formal redescription of Balaenopteroidea needs to be conducted with the clear inclusion of *E. robustus* within the family Balaenopteridae. Conflicting relationships represented by the variable support values of Nodes 6 and 7 can be explained by the missing data in *B.omurai*, as the clade *B. musculus* + *Balaenoptera edeni* + *B. borealis* is supported by 100% BS values in RAxML analyses using Dataset B (Supplementary Fig. S1 available on Dryad).

### Phylogeny and Evolution of Odontoceti

We find continued robust support for a monophyletic Odontoceti (Fig. 2), which is consistent with unique synapomorphies such as the lateral expansion of the maxilla coinciding with the development of echolocation (Geisler et al. 2014). Sperm whales (Physeteridae + Kogiidae) split from other extant odontocetes in the Latest Eocene ($\bar{x}= 34.13$ Ma; Fig. 3); however, there is no evidence of either stem or crown odontocetes present in the Eocene (Marx et al. 2016). Physeteridae and Kogiidae diverged from one another in the Late Oligocene or Early Miocene ($\bar{x}= 22.11$ Ma; 95% CI: 20.58–24.08; Supplementary Table S3 available on Dryad). This agrees with other earlier divergence analyses (McGowen et al. 2009; Steeman et al. 2009; Meredith et al. 2011; Hassanin et al. 2012). *Kogia* and *Physeter* have sometimes been included in the same family (Physeteridae), but the divergence between these genera is earlier than the diversification of Superfamily Delphinoidea, and these genera likely warrant placement in separate families. This deep divergence also coincides with evidence of the existence of fossil kogiids in the Early Miocene (Velez-Juarbe et al. 2015)*.*

*Platanista gangetica* is a freshwater odontocete found in the river systems of South Asia (Indus, Ganges, and Brahmaputra) and a relict species that is part of a lineage that was much more diverse in the past, with multiple fossils known from marine deposits (Geisler et al. 2011; Marx et al. 2016). Although we included *Platanista* sequences from only 72 genes, we recovered strong support for *P. gangetica* as the sister taxon of all other odontocetes excluding sperm whales (Fig. 2), placing them in a distinct clade from the other “river dolphins” (*Inia*, *Lipotes*, and *Pontoporia*). Some analyses of mainly mitochondrial data have united *Platanista* and Ziphiidae (Cassens et al. 2000; Hassanin et al. 2012), but analyses integrating significant nuclear-derived data have agreed with results presented here (McGowen et al. 2009; Steeman et al. 2009; Chen et al. 2011; Geisler et al. 2011; Meredith et al. 2011; Nikaido et al. 2001; Zhou et al. 2011).

Phylogenetic relationships among ziphiids are fully resolved and well-supported with all but three species not included in our analysis (*Mesoplodon hotaula*, *Mesoplodon traversii*, *Indopacetus pacificus*; Fig. 2). The strong resolution obtained here is in contrast to the most comprehensive analyses of ziphiids hitherto undertaken, which resulted in many weakly supported nodes (Agnarsson and May-Collado 2008; Dalebout et al. 2008, 2014; McGowen et al. 2009; Steeman et al. 2009). Ziphiids started to diversify in the Early or Middle Miocene ($\bar{x}= 15.61$ Ma; 95% CI: 13.65–17.79; Fig. 3), with *Berardius* as the most basal genus in the family, followed by *Tasmacetus*, *Ziphius*, *Hyperoodon*, and *Mesoplodon* (Fig. 2). Our results nest *Tasmacetus shepherdi*, a beaked whale with multiple functional teeth in both jaws of both sexes within a clade that has a reduced dentition of two to four mandibular teeth (Ellis and Mead 2017). We find no support for a traditional division of ziphiids into two subfamilies: Ziphiinae (*Berardius*, *Ziphius*, and *Tasmacetus*) and Hyperoodontinae (*Indopacetus*, *Hyperoodon*, and *Mesoplodon*), although the monophyly of *Hyperoodon* + *Mesoplodon* is well-supported. *Mesoplodon* (the most speciose genus of all cetaceans with 15 recognized species) experienced a rapid radiation beginning in the Late Miocene, with at least 13 species arising in the span of less than 5 Ma (Fig. 3). Within *Mesoplodon*, we find support for three major clades: the “bidens” lineage which contains *bidens, gingkodens*, *europaeus*, and *mirus*; the “layardii” lineage including *layardii*, *carlhubbsi*, and *bowdoini*; and the “hectori” lineage which includes *hectori*, *grayi*, *stejnegeri*, *densirostris*, *perrini*, and *peruvianus*. Although weakly supported in some analyses, the *layardii* clade and some species in the *hectori* clade have been recovered by either mtDNA, nuclear introns, or both (Dalebout et al. 2002, 2007, 2008, 2014; McGowen et al. 2009; Steeman et al. 2009); here, we find robust unequivocal evidence for their support. The *gingkodens* + *mirus* + *europaeus* clade is well-supported in analyses of both mt and ncDNA, and our results also place *bidens* in a clade with these species (Fig. 2). This finding differs from many previous studies in which *M. bidens* was placed in a basal position with respect to all other *Mesoplodon* species, although this arrangement received mostly weak support (Dalebout et al. 2008, 2014; McGowen et al. 2009; Steeman et al. 2009).

Previous molecular analyses differed as to the phylogenetic relationships among the remaining “river dolphin” species (*Inia*, *Pontoporia*, and *Lipotes*). In some molecular analyses, *Lipotes* was placed as the most basal taxon of the Delphinida (Delphinoidea + Iniidae + Pontporiidae + Lipotidae; Cassens et al. 2000; Hamilton et al. 2001) or weakly supported as sister to *Inia* + *Pontoporia* (Agnarsson and May-Collado 2008; McGowen et al. 2009; Steeman et al. 2009; Geisler et al. 2011; Hassanin et al. 2012). In this study, we find strong evidence that all three species form a distinct clade (Fig. 2), which originated in the Late Oligocene ($\bar{x}= 23.97$ Ma; 95% CI: 23.03–24.92). Evidence from previous analyses integrating molecular, morphological, and fossil data reveal that the two solely freshwater species, *Lipotes* and *Inia*, invaded freshwater separately, as they are more closely related to fossil taxa from marine sediments (Geisler et al. 2011).

Delphinoidea (Monodontidae + Phocoenidae + Delphinidae) is well-supported with Monodontidae more closely related to Phocoenidae, as noted in previous analyses (Waddell et al. 2000; Cassens et al. 2000; Agnarsson and May-Collado 2008; McGowen et al. 2009; Steeman et al. 2009; McGowen 2011; Zhou et al. 2011; Hassanin et al. 2012). Crown delphinoids originated in the Early Miocene ($\bar{x}= 19.78$ Ma; 95% CI: 18.81–20.76). Fossil lineages grouped in the “Kentriodontidae” have been tied to the early diversification of Delphinida and Delphinoidea, but revision of this group is in process (Murakami et al. 2014; Peredo et al. 2018). Both Crown Phocoenidae and Crown Monodontidae originated in the Late Miocene (Fig. 3). Within phocoenids, we strongly recovered *Neophocoena* as the most basal genus as well as a monophyletic *Phocoena*. The monophyly of *Phocoena* conflicts with multiple analyses that have placed *Phocoena phocoena* and *P. dalli* as sister species to the exclusion of other members of *Phocoena*; however, relationships between these species were usually weakly supported and/or dominated by mitochondrial data (Pichler et al. 2001; McGowen et al. 2009; Steeman et al. 2009).

Our phylogenomic reconstruction provided for a clear picture of the evolutionary relationships within Delphinidae, with high statistical support and agreement between analyses for most clades (Fig. 2). The mean age of Crown Delphinidae using the AR model ($\bar{x}=12.72$ Ma) is older by almost 3 Ma than the IR model ($\bar{x}= 9.86$ Ma), which is similar in age to some previous clock analyses (McGowen et al. 2009; Steeman et al. 2009; Slater et al. 2010; Hassanin et al. 2012). Most other divergences within the clade occur either shortly, thereafter, or in a somewhat simultaneous burst in the Late Miocene/Pliocene that corresponds to the major subfamilies (Fig. 3). Relationships within Delphinidae, the most speciose cetacean family, have been notoriously difficult to resolve, although several large-scale analyses in recent years have improved resolution markedly (McGowen et al. 2009; Steeman et al. 2009; McGowen 2011). We find continued support for three species at the base of Delphinidae (*Leucopleurus acutus*, *O. orca*, and *Lagenorhynchus albirostris*; Fig. 2), although their configuration differs from an earlier large-scale analysis of the group, which places *O. orca* as sister to the remaining delphinids to the exclusion of both *L. acutus* and *L. albirostris* (McGowen 2011). Neither *L. acutus* nor *L. albirostris* is closely related to the other former members of *Lagenorhynchus*, now included within the genus *Sagmatias* (*sensu*Leduc et al. 1999; Vollmer et al. 2019; Fig. 2). Exclusive of these three species, we find overwhelming support for three major clades that roughly correspond to previously identified subfamilies: Lissodelphininae, Globicephalinae (with the inclusion of *Grampus*, *Orcaella*, and *Steno*), and Delphininae (with the inclusion of *Sousa* and *Sotalia*).

Relationships within Lissodelphininae (*Lissodelphis, Sagmatias, Cephalorhynchus*) resemble those of previous studies using both mitochondrial and nuclear data (McGowen et al. 2009; McGowen 2011). We were unable to include three lissodelphinine species here (*Sagmatias cruciger*, *Cephalorhynchus eutropia*, *C. hectori*); however, *C. eutropia* and *C. hectori* are consistently allied with *Cephalorhynchus commersoni*, and *S. cruciger* is well established as the sister species to *S. australis* (Pichler et al. 2001; Harlin-Cognato and Honeycutt 2006; May-Collado and Agnarsson 2006; McGowen et al. 2009; Steeman et al. 2009; McGowen 2011; Banguera-Hinestroza et al. 2014; Vollmer et al. 2019). Both our concatenated and coalescence results imply that the current genera *Cephalorhynchus* and *Sagmatias* are paraphyletic and need further taxonomic revision. *Cephalorhynchus heavisidii* is more closely related to *S. australis* than to other members of *Cephalorhynchus* (May-Collado and Agnarsson 2006; McGowen 2011), and it is likely that *S. australis* (and *S. cruciger*) will need to be transferred to *Cephalorhynchus* pending more complete sampling.

We find overwhelming support for the inclusion of the genera *Orcaella*, *Steno*, and *Grampus* within the subfamily Globicephalinae, with *Orcaella* and *Steno* diverging from other globicephalines in the Late Miocene (Fig. 3). Previous analyses of nuclear data supported the alliance of these genera with what were traditionally called the “blackfish” (*Globicephala*, *Feresa*, *Pseudorca*, *Peponocephala*; Caballero et al. 2008; McGowen et al. 2008, 2009; Steeman et al. 2009; Banguera-Hinestroza et al. 2014). In addition, our results provide clear evidence that neither *Orcaella* nor the “blackfish” is closely allied to *Orcinus*; globicephalines had been linked to *Orcinus* in the past (Leduc et al. 1999), and some mtDNA analyses linked *Orcinus* and *Orcaella* (Leduc et al. 1999; Agnarsson and May-Collado 2008). Although *Steno* is overwhelmingly grouped with globicephalines based on nuclear data, complete mt genomes have strongly supported its sister relationship with *Sotalia* and alliance with Delphininae (Cunha et al. 2011; Vilstrup et al. 2011), demonstrating extreme mitonuclear discordance that may have resulted from ancient introgression of the mitochondrial lineage.

There has been difficulty in resolving the $\sim $14 currently recognized species within the subfamily Delphininae (*Tursiops*, *Stenella*, *Sousa*, *Sotalia*, *Lagenodelphis*, and *Delphinus*), likely due to rapid speciation and the documented presence of viable intergeneric hybrids in this group (Fig. 1; Perrin et al. 2013; Bérubé and Palsbøll 2018). In this study, we find high support for the polyphyly of *Stenella*, with *Tursiops*, *Delphinus*, and *Lagenodelphis* nested within the genus (Fig. 2), as suggested by multiple previous molecular studies using mtDNA, nuDNA, or both (Fig. 1; Leduc et al. 1999; Caballero et al. 2008; Kingston et al. 2009; McGowen et al. 2009; Steeman et al. 2009; Xiong et al. 2009; McGowen 2011; Amaral et al. 2012). However, our analysis shows high support for *Sotalia* and *Sousa* as the most basal delphinine genera, as well as strong support for at least three other lineages that differ from previous analyses. One lineage of dolphins contains species with a distinctive contrasting pattern of patches and stripes, a “striped dolphin” lineage: *Delphinus*, *Lagenodelphis*, *S. coeruleoalba*, *S. longirostris*, and *Stenella clymene*. The other two lineages include the spotted dolphins (*S. attenuata* + *S. frontalis*) and the bottlenose dolphins (*T. truncatus* and *T. aduncus*), with both forming a monophyletic group with respect to the “striped” dolphin lineage. These three clades were recovered by Amaral et al. (2012) using coalescence analyses of 13 nuclear loci and mtDNA (Fig. 1), but relationships among these clades differed based on method (Fig. 2), and inclusion of more data could change the patterns presented here. The monophyly of the “striped” dolphin lineage has morphological support from at least seven cranial characters including a rostrum which is dorsoventrally compressed distally, small temporal fossae compared with other delphinines, and a grooved or slightly grooved palate (Perrin et al. 1981). Perrin et al. (1987) noted similarities in characters of the spotted and bottlenose dolphins including external coloration and cranial characters such as a smooth palate and large temporal fossae. Many previous studies have suggested synonymizing *Lagenodelphis*, *Stenella*, *Tursiops*, and sometimes *Sousa* with *Delphinus* (Leduc et al. 1999; Caballero et al. 2008; McGowen et al. 2009; McGowen 2011; Perrin et al. 2013); however, a formal redescription has not been attempted due to the instability of relationships among phylogenetic studies. We suggest a less disruptive option by referring all species in the “striped” lineage (*L. hosei*, *S. coeruleoalba*, *S. clymene*, *S. longirostris*) to *Delphinus*, and retaining *Stenella* for the spotted dolphins (*S. attenuata* is the type species of *Stenella* [Perrin et al. 1987]) and *Tursiops* for the bottlenose dolphins.

Recently the validity of the species *Delphinus capensis*, the “long beaked common dolphin”, has been called into question (Natoli et al. 2006; Kingston et al. 2009; Cunha et al. 2015; Farías-Curtidor et al. 2017), and the Society of Marine Mammalogy Committee on Taxonomy has recommended the use of *D. delphis* for all members of the genus (as used here) until further detailed analyses can be completed (Society for Marine Mammalogy Committee on Taxonomy 2017). The subspecies *bairdii* and *tropicalis*, both included in this study, had been referred to *capensis*, as they represented morphologically long-beaked forms (Heyning and Perrin 1994; Jefferson and Van Waerebeek 2002). In agreement with Cunha et al. (2015), we find that the putative species *D. capensis* is paraphyletic with respect to *D. delphis*, although only four individuals of the genus are represented here. We find that the long-beaked dolphin of the Indian Ocean (subspecies *tropicalis* previously included within *D. capensis* [Jefferson and Van Waerebeek 2002]) is more closely related to the representative short-beaked *D. delphis delphis* from the UK included here than either are to the long-beaked-type from California (*D. delphis bairdii*; 79929, 108471; Fig. 2).

One other difficult issue within Delphininae is the putative hybrid origin of the species *Stenella clymene.* Here, *S. clymene* is unequivocally well-supported as the sister species of *S. longirostris* based on substantial genomic data (Fig. 2). *Stenella clymene* was redescribed by Perrin et al. (1981), where it was noted that its external characteristics resembled *S. longirostris* but its skull resembled *S. coeruleoalba*. Molecular analyses of cytochrome $b$ showed that *S. clymene* and *S. coeruleoalba* grouped together, adding support to a potential hybrid origin (Leduc et al. 1999), and subsequent analyses with mtDNA have allied *S. clymene* and *S. coeruleoalba* (Leduc et al. 1999, May-Collado and Agnarsson 2006; McGowen et al. 2009). Contrary to these findings, nuclear DNA in the form of AFLPs grouped *S. clymene* strongly with *S. longirostris* (Kingston et al. 2009; Fig. 1). Amaral et al. (2014) sequenced multiple individuals of all three species and showed that the cytochrome $b$ sequence of most individuals of *S. clymene* were more closely related to *S. coerueloalba*, but others were closer to *S. longirostris*; however, nuclear DNA from five loci showed little differentiation between the three taxa and could easily be explained by ancestral polymorphism. Our study sequenced only two individuals of *S. clymene*, but whole genome-scale sequencing of multiple representatives of all three species will likely be needed to properly address the question of its potential hybrid origin.

## Conclusion

We targeted and assembled 3191 protein-coding genes from 68 species of cetaceans, two hippopotamids and three ruminants new to this study, and combined them with 18 existing genomes to produce the most comprehensive phylogenetic tree of cetaceans to date, in terms of the intersection of sequence data (38,167 exons; >6.5 million bp) and species (77 out of 89 total). Every node was well-resolved, including those within the problematic Delphinidae (true dolphins), although three nodes within the family differed between concatenated and coalescence analyses. Our results give clarity to a long debate over the contentious relationships among species currently contained within *Stenella*, *Lagenodelphis*, *Delphinus*, and *Tursiops*. Further analyses will seek to include the remaining 12 species; however, some of these were only recently split off from taxa represented here (e.g., *Sotalia fluviatilis*, *Sousa plumbea*, *S. sahulensis*), or are incredibly rare and represented by few specimens (*Mesoplodon traversii*, *M. hotaula*). Cetaceans are well-represented in the fossil record, and further studies will combine these new data with morphological and fossil data to produce a holistic view of cetacean evolution.

## Supplementary Material

Data available from the Dryad Digital Repository: https://doi.org/10.5061/dryad.jq40b0f

## Appendix

**Table A1.** List of tissue sampled and taxa new to this study

Species	Common name	ID	Location	Lending institution	Institution of origin	Origin ID
Cetacea						
*Balaenoptera bonaerensis*	Antarctic minke whale	199219648	Tasmania, Australia	AAD		
*Balaenoptera borealis*	Sei whale	SW2012/413	Northumberland, England	IOZ		
*Balaenoptera (edeni) edeni*	Bryde’s whale	1380856971	Tasmania, Australia	AAD		
*Balaenoptera (edeni) edeni*	Bryde’s whale	Z66737	Off Sinaloa, Mexico	SWFSC		
*Balaenoptera musculus*	Blue whale	Z49099	California, USA	SWFSC		
*Balaenoptera physalus*	Fin whale	SW1995-105	Kent, England	IOZ		
*Berardius arnuxii*	Arnoux’s beaked whale	Z9128	New Zealand	SWFSC	NZCeTA	BAR02
*Berardius bairdii*	Baird’s beaked whale	Z76728	West Coast, USA	SWFSC		
*Caperea marginata*	Pygmy beaked whale	Z5990	New Zealand	SWFSC		
*Cephalorhynchus commersonii*	Commerson’s dolphin	Z40	Captive	SWFSC		
*Cephalorhynchus heavisidii*	Heaviside’s dolphin	Z7320	Yzerfontein, South Africa	SWFSC	PBB	9622
*Delphinapterus leucas*	Beluga	Z55860	Alaska, USA	SWFSC	ADFG	BB2006-44
*Delphinus delphis bairdii*	N. Pac. long-beaked common dolphin	Z79929	California, USA	SWFSC		
*Delphinus delphis bairdii*	N. Pac. long-beaked common dolphin	Z108471	California, USA	SWFSC		
*Delphinus delphis delphis*	Short-beaked common dolphin	SW1999-92	Devon, England	IOZ		
*Delphinus delphis tropicalis*	Indo-Pacific common dolphin	Z4525	Indian Ocean, off Oman	SWFSC		
*Eschrichtius robustus*	Gray whale	Z133943	California, USA	SWFSC		
*Eubalaena australis*	Southern right whale	TAS1201	Tasmania, Australia	AAD		
*Eubalaena glacialis*	North Atlantic right whale	Z13086	Massachusetts, USA	SWFSC		
*Eubalaena japonica*	North Pacific right whale	Z43864	Alaska, USA	SWFSC		
*Feresa attenuata*	Pygmy killer whale	Z145402	Eastern North Pacific	SWFSC		
*Globicephala macrorhynchus*	Short-finned pilot whale	Z39091	California, USA	SWFSC		
*Globicephala melas*	Long-finned pilot whale	SW1997-162	Northumberland, England	IOZ		
*Grampus griseus*	Risso’s dolphin	SW1992-213	Dyfed, Wales	IOZ		
*Hyperoodon planifrons*	Southern bottlenose whale	Z9120	New Zealand	SWFSC	NZCeTA	HPL01
*Hyperoodon ampullatus*	Northern bottlenose whale	SW2006-40	London, England	IOZ		
*Inia geoffrensis*	Amazon river dolphin	Z505	Acre, Brazil	SWFSC	USNM	571366
*Kogia sima*	Dwarf sperm whale	Z12696	Florida, USA	SWFSC		
*Kogia breviceps*	Pygmy sperm whale	SW1997-159	Pembrokeshire, Wales	IOZ		
*Lagenodelphis hosei*	Fraser’s dolphin	Z452	North Pacific	SWFSC	USNM	500354
*Lagenodelphis hosei*	Fraser’s dolphin	Z30470	Hawaii, USA	SWFSC		
*Lagenorhynchus (Sagmatias)* *australis*	Peale’s dolphin	Z4926	Cabo Espiritu Santo, Chile	SWFSC		
*Lagenorhynchus (Sagmatias) obliquidens*	Pacific white-sided dolphin	Z31902	California, USA	SWFSC		
*Lagenorhynchus (Sagmatias) obscurus*	Dusky dolphin	Z2318	Peru	SWFSC		
*Lagenorhynchus (Leucopleurus) acutus*	Atlantic white-sided dolphin	SW1998-90	North Yorkshire, England	IOZ		
*Lagenorhynchus albirostris*	White-beaked dolphin	SW1999-201A	Humberside, England	IOZ		
*Lissodelphis borealis*	Northern right-whale dolphin	Z113034	California, USA	SWFSC		
*Lissodelphis peronii*	Southern right-whale dolphin	LPER020904	Tasmania, Australia	AAD		
*Mesoplodon bidens*	Sowerby’s beaked whale	SW1998-81	Lincolnshire, England	IOZ		
*Mesoplodon bowdoini*	Andrews’s beaked whale	Z9109	New Zealand	SWFSC	NZCeTA	ZCA01
*Mesoplodon carlhubbsi*	Hubbs’s beaked whale	Z1563	California, USA	SWFSC		
*Mesoplodon densirostris*	Blainville’s beaked whale	SW1993-78	Dyfed, Wales	IOZ		
*Mesoplodon europaeus*	Gervais’s beaked whale	Z7444	Florida, USA	SWFSC		
*Mesoplodon ginkgodens*	Ginkgo-toothed beaked whale	MginNZ03	Taranaki, New Zealand	NZCeTA		
*Mesoplodon grayi*	Gray’s beaked whale	210210	Tasmania, Australia	AAD		
*Mesoplodon hectori*	Hector’s beaked whale	Z9115	New Zealand	SWFSC		
*Mesoplodon layardii*	Strap-toothed whale	1763273011	Tasmania, Australia	AAD		
*Mesoplodon mirus*	True’s beaked whale	Z4972	New Jersey, USA	SWFSC	USNM	504612
*Mesoplodon perrini*	Perrin’s beaked whale	Z4976	California, USA	SWFSC	USNM	504259
*Mesoplodon peruvianus*	Pygmy beaked whale	Z23629	California, USA	SWFSC		
*Mesoplodon stejnegeri*	Stejneger’s beaked whale	Z107244	Alaska, USA	SWFSC		
*Monodon monoceros*	Narwhal	Z8293	Uummannaq, Greenland	SWFSC	GINR	GF16213
*Neophocaena phocaenoides*	Indo-Pacific finless porpoise	Z61334	Hong Kong	SWFSC		
*Orcaella brevirostris*	Irrawaddy dolphin	Z7205	Mekong River, Laos	SWFSC		
*Orcaella heinsohnii*	Australian snubfin dolphin	Z2907	Queensland, Australia	SWFSC		
*Peponocephala electra*	Melon-headed whale	Z41110	Hawaii, USA	SWFSC		
*Phocoena dioptrica*	Spectacled porpoise	Z981	Est. Las Violetas, Argentina	SWFSC		
*Phocoena phocoena*	Harbor porpoise	SW2000-104	Ceredigion, Wales	IOZ		
*Phocoena spinipinnis*	Burmeister’s porpoise	Z1092	Peru	SWFSC		
*Phocoenoides dalli*	Dall’s porpoise	Z4824	California, USA	SWFSC		
*Pontoporia blainvillei*	Franciscana	Z7349	Necochea, Argentina	SWFSC		
*Pseudorca crassidens*	False killer whale	Z123188	Molokai, Hawaii, USA	SWFSC	KW	KW2010019
*Sotalia guianensis*	Guiana dolphin	Z9837	Natal, Brazil	SWFSC		
*Sousa chinensis*	Indo-Pacific humpback dolphin	Z77289	Hong Kong	SWFSC	TJ	HKB42
*Stenella attenuata*	Pantropical spotted dolphin	Z18473	Tropical Eastern Pacific	SWFSC		
*Stenella attenuata*	Pantropical spotted dolphin	Z38219	Tropical Eastern Pacific	SWFSC		
*Stenella clymene*	Clymene dolphin	Z1724	Gulf of Mexico	SWFSC		
*Stenella clymene*	Clymene dolphin	Z1726	Gulf of Mexico	SWFSC		
*Stenella coeruleoalba*	Striped dolphin	SW2000-22	Devon, England	IOZ		
*Stenella frontalis*	Atlantic spotted dolphin	Z7782	NW Atlantic	SWFSC		
*Stenella frontalis*	Atlantic spotted dolphin	Z7784	NW Atlantic	SWFSC		
*Stenella longirostris*	Spinner dolphin	Z16012	Tropical Eastern Pacific	SWFSC		
*Stenella longirostris*	Spinner dolphin	Z24923	Tropical Eastern Pacific	SWFSC		
*Steno bredanensis*	Rough-toothed dolphin	Z18431	Tropical Eastern Pacific	SWFSC		
*Steno bredanensis*	Rough-toothed dolphin	Z116871	Saipan, N. Marianas Islands	SWFSC	PIFSC	PIC130720.01B
*Tasmacetus shepherdi*	Shepherd’s beaked whale	Z4971	Chubut, Argentina	SWFSC	USNM	484878
*Tursiops aduncus*	Indo-Pacific bottlenose dolphin	Z79924	Berau Archipelago, Indonesia	SWFSC	YK-RASI	TADU080423
*Ziphius cavirostris*	Cuvier’s beaked whale	SW2002-222	Norfolk, England	IOZ		
Hippopotamidae						
*Hippopotamus amphibius*	Common hippopotamus		Captive	CZ		
*Choeropsis liberiensis*	Pygmy hippopotamus	WHMO71/0546/50	Captive	ZSL		
Bovidae						
*Oryx leucoryx*	Arabian oryx	zm693/05 4369	Captive	Taipei Zoo		
*Tragelaphus eurycerus*	Bongo	20080367M10	Captive	ZSL		
*Gazella arabica*	Arabian gazelle	zm634/0821/7/08	Captive	Taipei Zoo		

AAD, Australian Antarctic Division; ADFG, Alaska Department of Fish and Game; CZ, Copenhagen Zoo; GINR, Greenland Institute of Natural Resources; IOZ, Institute of Zoology, Zoological Society of London; KW, Kristi West, University of Hawai’i; NZCeTA, New Zealand Cetacean Tissue Archive; PBB, Peter Best, South African Museum; PIFSC, NOAA, Pacific Islands Fisheries Science Center; SWFSC, NOAA, Southwest Fisheries Science Center; TJ, Thomas Jefferson, Clymene Enterprises; USNM, Smithsonian National Museum of Natural History; YK-RASI, Yayasan Konservasi RASI; ZSL, Zoological Society of London.
